# How the Mode of Delivery Is Influenced by Patient’s Opinions and Risk-Informed Consent in Women with a History of Caesarean Section? Is Vaginal Delivery a Real Option after Caesarean Section?

**DOI:** 10.3390/jcm13154393

**Published:** 2024-07-27

**Authors:** Ionut Marcel Cobec, Andreas Rempen, Diana-Maria Anastasiu-Popov, Anca-Elena Eftenoiu, Aurica Elisabeta Moatar, Tania Vlad, Ioan Sas, Vlad Bogdan Varzaru

**Affiliations:** 1 ANAPATMOL Research Center, Faculty of Medicine, “Victor Babes” University of Medicine and Pharmacy Timisoara, 300041 Timisoara, Romania; 2 Clinic of Obstetrics and Gynecology, Klinikum Freudenstadt, 72250 Freudenstadt, Germany; 3 Clinic of Obstetrics and Gynecology, Diakoneo Diak Klinikum Schwäbisch Hall, 74523 Schwäbisch Hall, Germany; 4 Department of Medical Genetics, “Carol Davila” University of Medicine and Pharmacy, 014461 Bucharest, Romania; 5 Clinic of Internal Medicine-Cardiology, Klinikum Freudenstadt, 72250 Freudenstadt, Germany; 6 Faculty of Medicine, “Victor Babes” University of Medicine and Pharmacy Timisoara, 300041 Timisoara, Romania; 7 Department of Obstetrics and Gynecology, “Victor Babes” University of Medicine and Pharmacy, 300041 Timisoara, Romania; 8 Doctoral School, Faculty of Medicine, “Victor Babes” University of Medicine and Pharmacy Timisoara, 300041 Timisoara, Romania

**Keywords:** caesarean section, vaginal birth, vaginal delivery, pregnancy

## Abstract

**Background/Objectives**: In recent years, there has been a noticeable increase in the rates of caesarean section (CS), being one of the most commonly performed surgical procedures. For the following pregnancy, the previous CS represents the backbone of the risks and complications, such as uterine scar formation, uterine rupture, massive bleeding, and serious negative outcomes for both the mother and child. Our study followed patients with a history of CS from the birth planning prenatal check-up to delivery. **Methods**: We reviewed the records of 125 pregnant women with previous CS who presented in the third trimester for a prenatal check-up and completed our questionnaire from March 2021 to April 2022 in the Clinic of Obstetrics and Gynecology, Diakoneo Diak Klinikum Schwäbisch Hall, Germany. **Results**: Before the prenatal check-up, 74 patients (59.2%) preferred vaginal delivery (VD), while 51 (40.8%) preferred CS. After discussing birth planning with the obstetrician, 72 women (57.6%) decided upon VD, while 53 (42.4%) preferred CS. Ultimately, 78 (62.4%) of women gave birth through CS (either planned or by medical necessity) and 47 (37.6%) gave birth vaginally (either natural or per vacuum extraction). **Conclusions**: VD for patients with CS in their medical history is a real option. The patient must be well informed about the risks and benefits of the medical situation and should be empowered and supported on their chosen mode of delivery, which should be respected.

## 1. Introduction

In recent years, it has generally been noticed that caesarean section (CS) rates are increasing [1,2], being one of the most frequently performed surgical procedures [3]. For example, in the United States, the frequency of CS has seen an increase over the past two decades, to the extent that one in three women giving birth undergo a CS today, a trend seen in many other developed and developing countries as well [2,4]. On the other hand, recent years show a significant improvement in the success rate and safety of CS [5]. For the subsequent pregnancy, the previous CS represents the backbone of the risks and complications, such as uterine scar formation, uterine rupture, massive bleeding, and serious negative outcomes for mother and child [5].

The maternal morbidity of CS is higher than that of vaginal delivery (VD), even in the presence of a history of CS [6]. Maternal risks associated with CS compared to VD include haemorrhagic and thromboembolic complications, organ injury during surgery, unplanned hysterectomy, prolonged hospitalization, more readmissions, and greater maternal mortality [2,7]. Other maternal complications include chronic pain, scarring, and intestinal adhesions. Furthermore, subsequent pregnancies following a previous caesarean delivery may be at an increased risk of unexplained stillbirths, placental abnormalities, and repeated caesarean delivery [2,7]. Meanwhile, VD may also present complications compared to CS, such as pelvic floor disorders [2,8].

Actual medical concepts and health awareness play a key role in advocating for VD [5]. VD typically involves a shorter hospitalization period compared to CS, along with a quicker and more efficient postpartum recovery, and a generally good outcome for both mother and child [5,9].

In subsequent pregnancies, a history of CS poses risks for uterine rupture, uterine scar dehiscence, abnormal placental implantation, and placental abruption [10]. The literature reports uterine rupture and neonatal asphyxia as the primary risks associated with VD after CS and the failure of vaginal trial delivery increases postpartum haemorrhage and hysterectomy risk [5]. Based on these risks, many patients with CS in their medical history opt for CS in the following pregnancies. According to the literature, the second CS has risks such as damage to the uterine tissue and increased postpartum haemorrhage [5].

Some studies report that pregnant women with a history of CS make decisions regarding their mode of delivery based on their educational level and their understanding of the advantages and disadvantages of CS, or fear of the pain associated with vaginal childbirth. Many women who attempted VD after CS ultimately opted for CS due to a lack of confidence [1,5,11,12,13].

Among the factors with a negative effect on VD rates after CS are repeated indication for CS, interval time from prior CS, advanced maternal age, elevated maternal body mass index (BMI), maternal height, advanced gestational age, pre-eclampsia, and labour induction [11]. Rare conditions, like uterus abnormalities, should be diagnosed as early as possible [1].

Repeated delivery via CS is associated with increased risks of hysterectomy, bowel or bladder injury, blood transfusion, placenta previa or placenta accreta, and increased medical costs [11].

Among the fetal factors, which play a key role in choosing CS to end delivery, are fetal macrosomia, twin pregnancy, fetal abnormalities, and fetal distress [3,5].

Currently, the VD rate has been improved by the use of epidural anesthesia, which effectively reduces labour pain [9].

Induced labour with oxytocin increases the risk of uterine rupture compared to spontaneous labour [9].

It is very important that obstetricians and pregnant women carefully consider the maternal and neonatal risks and benefits associated with VD after CS or CS after CS. The purpose of the present study is to determine the influence of patients’ perceptions and risk-informed consent on the mode of delivery in women with previous CS. This question has also been raised in the German guidelines for CS [14].

## 2. Materials and Methods

This study represents a questionnaire-based study, which used anonymized data collected over a period of one year, pertaining to 125 pregnant women with a history of CS who presented in the third trimester for a prenatal check-up and completed our questionnaire from March 2021 to April 2022 in the Clinic of Obstetrics and Gynecology, Diakoneo Diak Klinikum Schwäbisch Hall, Germany. During their visit, patients received a fetal ultrasound and discussed, in detail, the advantages and disadvantages of VD versus CS, as well as any additional risks associated with a history of CS. The patients were informed about the questionnaire by a doctor through written and verbal explanations.

All patients were managed according to the current German national guidelines for vaginal delivery after caesarean section [14,15]. Patients with a history of CS and a fetus in cephalic presentation are offered the option of vaginal delivery provided they express informed consent. Exclusions are limited to cases with definitive contraindications to VD.

The gathered data relate to the patients from the moment of the prenatal check-up to delivery.

The following data were collected from our database: maternal age, gravidity, parity, notable diagnoses of the patient, the indication for the previous (first) CS, gestational age at the moment of birth planning, the actual mode of delivery and the indication for it, gestational age at delivery, use of labour induction and the indication for it, complications of VD, and neonatal parameters (weight; Apgar scores at 1, 5, and 10 min postpartum; fetal blood pH).

The questionnaire comprised their preferences and justifications regarding the delivery methods, before and after the prenatal consultation.

For statistical analysis and data visualization, we used the software IBM SPSS Statistics 20 software and Microsoft Office Excel 2019.

## 3. Results

In the observed year, we registered 1491 singleton deliveries and 47 twin deliveries in our clinic and a general CS rate of 29.5%. Overall, 14.2% were elective planned CSs and 15.3% CSs were performed during labour by medical necessity. A total of 15.3% of all registered deliveries in our clinic in the observed period were patients with at least one CS in their medical history. A total of 31.1% of those patients delivered vaginally and 68.9% of those patients delivered through CS.

A total of 125 patients were included in our study. The mean maternal age was 32.51 years (SD = 4.442), while the youngest patient was 20 and the oldest was 45 years old. The birth planning prenatal check-up was performed between 32 + 6 weeks of pregnancy and 39 + 1 weeks of pregnancy. A total of 56.8% of the patients were gravida 2, 29.6% were gravida 3, 8.8% were gravida 4, 4.0% were gravida 5, and 0.8% were gravida 8. A total of 84.8% of the patients had parity 1, 14.4% had parity 2, and 0.8% had parity 3.

### 3.1. Questionnaire Response Analysis

**Figure 1:**
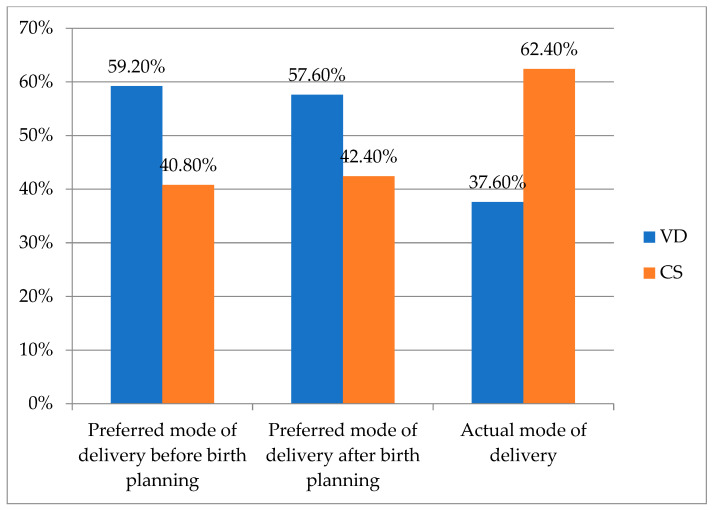
Comparison between preferred mode of delivery before birth planning, preferred mode of delivery after birth planning, and actual mode of delivery. CS = caesarean section; VD = vaginal delivery.

The registered preferences of the patients before the prenatal birth planning check-up show that 74 patients (59.2%) preferred VD, while 51 (40.8%) preferred CS (Figure 1).

A total of 74 women expressed a preference for VD before the prenatal check-up, stating the following reasons: “VD is natural” (54.1%), want to experience VD (16.2%), had a traumatic experience during CS (8.1%), “VD is better for the body” (6.8%), “CS is painful” (4.1%), faster hospital discharge (2.7%), “VD is more pleasant” (1.4%), and faster recovery (1.4%), while 5.4% did not specify a reason.

The 51 women who preferred CS before the prenatal check-up stated the following reasons: traumatic birth experience (27.5%), fear of VD (19.6%), “CS is more comfortable” (17.6%), fear of complications (9.8%), scar pain (3.9%), shorter interval between pregnancies (3.9%), herniated disc (2.0%), desire for sterilisation during the CS (2.0%), history of myoma enucleation (2.0%), maternal age (2.0%), and epilepsy in their medical history (2.0%), while 7.8% did not specify a reason.

After discussing birth planning with the obstetrician, 72 women (57.6%) decided upon VD, while 53 (42.4%) preferred CS (Figure 1).

A total of 10 women also had a midwifery consultation. One of them had already discussed birth planning with the physician and consistently opted for VD. A total of seven patients preferred VD and three patients preferred CS, before and after the midwifery consultation.

Table 1 shows that two patients changed their preferred mode of delivery from vaginal birth to CS during birth planning. One case was of fetal macrosomia and the other was of trisomy 21. Accordingly, both patients gave birth through CS. No patients who preferred CS before birth planning changed their preference to VD after birth planning.

A McNemar test showed that the proportions of the preferred mode of delivery before and after birth planning medical check-up were not significantly different (*p* = 0.5).

We performed independent sample *t*-tests to compare maternal ages between women who preferred VD or CS (before and after birth planning discussion) and we found no significant difference (*p* = 0.779 before the prenatal check-up and *p* = 0.631 after the prenatal check-up).

### 3.2. Actual Mode of Delivery

CS was performed in 78 cases (62.4%), of which 50 (40.0%) were represented by planned CS, while the other 28 (22.4%) were represented by CS performed during labour by medical necessity. Out of forty-seven women (37.6%) who delivered vaginally, forty-two women (33.6%) delivered through non-instrumental VD, while five (4.0%) required vacuum extraction (Figure 1). A total of 96 women (76.8%) gave birth according to their preferred mode of delivery after the birth planning discussion.

Out of seventy-two patients who decided upon delivering vaginally after discussion with the obstetrician, twenty-seven (37.5%) delivered through CS (seven patients planned CS; twenty patients had CS performed during labour by medical necessity) and forty-five delivered vaginally. Of the fifty-three patients who decided upon CS, two (3.77%) patients delivered naturally. These patients preferred CS before as well as after discussion with the obstetrician. One of them delivered naturally because of stillbirth. Of the other fifty-one patients who decided upon CS, forty-three delivered through planned CS and eight through CS performed during labour by medical necessity (Table 2).

### 3.3. CS Indications: Past and Present

The most common indication for the prior CS was breech presentation (22.4%), followed by pathological cardiotocography (CTG) and failure to progress in labour, each representing 21.6%. The latter can be divided into stalled active labour phase (11.2%) and stalled early labour phase (10.4%). CS was performed because of the patient’s preference in 9.6%. Other less common indications were multiple births (3.2%), intra-amniotic infection (2.4%), HELLP syndrome (2.4%), pre-eclampsia (2.4%), unsuccessful labour induction (2.4%), physician’s decision (2.4%), and placental abruption (1.6%). Finally, the most uncommon indications for the prior CS, with one case (0.8%) each, were intrauterine growth restriction (IUGR) with pathological Doppler ultrasound with centralization of blood circulation, history of third-degree perineal tear, history of postpartum neonatal death of unknown cause, heavy vaginal bleeding, macrosomia, gastroschisis, epilepsy, history of shoulder dystocia, and history of myoma enucleation. In one case (0.8%) the indication for the prior CS was unknown.

In what concerns the indication for the actual mode of delivery, 54, representing 69.2% of the total CS, were performed at the request of the patient with a history of CS. In seven pregnancies (9.0%) CS was indicated in the case of pathological CTG, and in six pregnancies (7.7%) CS was indicated in case of failure to progress in labour (five stalled active labour phase; one stalled early labour phase). Breech presentation represented an indication for CS in two cases (2.6%). CS was indicated due to macrosomia in two cases (2.6%). Other less common indications for CS, with one case (1.3%) each, consisted of occiput posterior position, intra-amniotic infection, unsuccessful labour induction, inguinal pain, suspicion of uterine rupture, history of myoma enucleation, and epilepsy (Table 3).

For the patients who received CS, we explored the relationship between the preferred mode of delivery after the prenatal check-up, the type of CS (planned or CS performed during labour by medical necessity), and the indication for it. We aimed to compare the indications for the current CS between patients who decided upon CS and patients who decided upon VD.

In the first group of fifty-one patients, forty-five patients underwent CS at the request of the patient with a history of CS (thirty-eight planned CS; seven CS performed during labour by medical necessity), two patients underwent CS because of breech presentation (planned CS), one patient underwent CS because of fetal macrosomia (planned CS), one underwent CS because of inguinal pain (CS performed during labour by medical necessity), one underwent CS because of history of myoma enucleation (planned CS), and one underwent CS because of epilepsy (planned CS) (Table 4).

In the second group of twenty patients, 33.3% delivered through CS at the request of the patient with a history of CS (six planned CS; three CS performed during labour by medical necessity), 25.9% delivered through CS because of pathological CTG (CS performed during labour by medical necessity), 22.2% delivered through CS because of failure to progress in labour (CS performed during labour by medical necessity), 3.7% delivered through CS because of occiput posterior position (CS performed during labour by medical necessity), 3.7% delivered through CS because of intra-amniotic infection (CS performed during labour by medical necessity), 3.7% delivered through CS because of macrosomia (planned CS), 3.7% delivered through CS because of unsuccessful labour induction (CS performed during labour by medical necessity), and 3.7% delivered through CS because of suspicion of uterine rupture (CS performed during labour by medical necessity) (Table 5).

We aimed to investigate whether the indications for the current CS performed due to medical necessity in patients preferring VD after the prenatal check-up correlated with the indications for the prior CS (Table 6).

### 3.4. Challenges and Complications of VD

Out of 75 patients who delivered either naturally, through vacuum extraction, or CS performed during labour by medical necessity, 14 (18.7%) required labour induction using dinoprostone vaginal gel. The indications for labour induction were as follows: overdue pregnancy +10 days (six cases), intrauterine growth restriction (IUGR) (four cases), gestational diabetes requiring insulin (one case), overdue pregnancy +7 days with gestational diabetes controlled by diet and exercise (one case), stillbirth (one case), and large for gestational age (LGA) (one case). The five vacuum extractions were indicated by pathological CTG.

We registered the medical complications that occurred in our studied group. For vacuum extraction, we registered one third-degree perineal tear. A total of 18 of 42 patients (42.9%) who delivered naturally (non-instrumental) had one or more complications. For each complicated case, we registered one or more complications. The most frequent complication of natural delivery was pathological CTG (eleven cases), followed by green amniotic fluid (four cases), nuchal cord (four cases), and one case for each of the following: third-degree perineal tear, difficult fetal shoulder delivery, placental abruption, and incomplete placenta delivery with curettage and/or bleeding.

### 3.5. Neonatal Outcomes

Gestational age at the moment of delivery ranged from 36 + 2 weeks of pregnancy to 41 + 6 weeks of pregnancy. For the following neonatal parameters, one case was excluded because of intrauterine fetal death. The mean newborn weight at delivery was 3468.95 g (SD = 477.399), ranging from 2100 g to 5030 g. The mean fetal blood pH at delivery was 7.27 (SD = 0.072), with a minimum registered value of 7.07 and a maximum of 7.47. We mention that one child had a blood pH under 7.10 (represented by 7.07) and nineteen newborn children had a pH between 7.10 and 7.19, while the other one hundred and four, representing 83.9% of the viable newborns, had a blood pH of at least 7.20.

We registered nine cases with a 1 min Apgar score lower than 8 (three cases of delivery through planned CS, five cases of delivery through CS performed during labour by medical necessity, and one vacuum extraction). The minimum 1 min Apgar score was 3, while the mean was 8.73 (SD = 1.127). At 5 min after birth, we registered one case with an Apgar lower than 8, i.e., a score of 6, delivered through vacuum extraction. The mean 5 min Apgar score was 9.72 (SD = 0.657). At 10 min after birth, there was no Apgar score under 8. The mean 10 min Apgar score was 9.94 (SD = 0.264). We noticed an Apgar score of 3/6/8 in a case of delivery per vacuum extraction.

An independent sample T-test was performed to compare the 5 min Apgar scores between newborns delivered through CS or VD and found no significant difference between the two groups (*p* = 0.401).

## 4. Discussion

The literature describes multiple factors that contribute to the rising CS rates. These factors vary among clinicians, midwives, women, cultures, and health systems. Interventions for reducing unnecessary CS have been widely studied [16,17,18,19]. Examples of such interventions include varied methods of labour induction, labour support, external cephalic version, and complementary therapies for pain relief. Conversely, interventions associated with an increased rate of CS include continuous CTG during labour, intermittent auscultation of the fetal heart with Doppler or intermittent CTG compared to Pinard stethoscope, and incentives linked directly with the initiation and frequency of antenatal care [20].

Studies show that large influences on the decision-making of clinicians are obstetrical experience, personal belief, comfort, informed consent, legal liability, and the perception that CS is either safer than or as safe as VD [21,22,23,24].

When it comes to the factors that influence women in decision-making, very important topics are freedom of choice, personal rights, and consumer centricity in healthcare. If a woman can choose to have plastic surgery, then she can choose to have a CS [25,26].

A Swedish study shows that the primary reasons for an elective CS in 1992 were breech presentation, abnormal fetal position, and uterine factors, whereas, in 2005, the primary reason was fear of childbirth in the absence of medical indications [26].

Other reasons for deciding upon CS are a family history of difficult labours, the presence of an underlying medical condition, age, social influences (primarily family and friends), pain or a bad outcome associated with VD, and traumatic births. In addition to these reasons, women in China also choose CS because of their husbands’ preference, the possibility to pick a lucky day, ethnic minority, age, and fertility treatment. The ideal number of children is also a factor in the decision-making process regarding childbirth. Women planning to have at least two children were less likely to prefer CS than those planning to have only one child. The ideal number of children reflects personal fertility intentions [27,28,29].

In about 30% of CSs performed in the developed world, the primary indication is the decision to decline VD after CS [30]. Women with prior CS choose a second CS mostly because of personal beliefs, previous birth experiences, the need for control, and practical considerations. VD after CS is associated with uterine rupture; however, this represents a 0.2% risk and when balanced against the morbidity associated with multiple CSs. VD after CS is considered a safe and valid choice [31,32,33,34].

The present study was performed in a perinatal level 1 clinic according to the German health system classification of obstetric clinics, where the CS rate is lower than Germany’s reported 31.8% [15,33]. Pregnant patients with a history of CS, who present relative or absolute CS indication or wish to have an elective CS, are invited to an ambulatory consultation and discussion with our consultant obstetrician regarding birth planning. The consultations in our study took place between 32 + 6 and 39 + 1 weeks of gestation, preferably with 35 or 36 weeks of gestation. We asked the patients to fill in a questionnaire with their wishes and reasons regarding childbirth, before and after the ambulatory visit. The study investigated the natural dynamic between patient birth preferences and actual outcomes. Its focus was on the factors that influence decision-making and complications that can occur during and after childbirth.

The policy of our clinic is oriented towards promoting natural birth. We facilitate continuous one-to-one support during labour, if wanted, as well as various methods for labour induction and acupuncture during labour. These discussions are led mostly by the same obstetrician, with experience in perinatal care. Of the 125 patients that came to the ambulatory, 59.2% preferred VD and 40.8% CS before the prenatal check-up. After the discussion with the obstetrician, two patients changed their minds from VD to CS, so that, in the end, 42.4% decided upon CS. Of the fifty-three women who decided upon CS, fifty-one received CS (forty-three planned; eight by medical necessity), while two gave birth vaginally (one stillbirth; one advanced labour).

In our study, we observed that out of the total patient cohort, only 10 patients opted for an additional consultation with a midwife. Within this subset of patients, none of the women exhibited a shift in their perspectives following the consultation with the midwife.

All patients who discussed with the obstetrician and preferred CS from the beginning maintained their decision after the consultation. None of them decided to give birth naturally. Only the opposite was observed, two patients who initially expressed a preference for VD changed their minds after the discussion with the obstetrician. There was no significant difference between the preferences before and after the prenatal medical check-up. Thus, our study suggests that most women come to the consultation having already decided on the mode of delivery and this decision is very often independent of the risk-informed discussion.

There is insufficient data regarding the importance women attribute to the outcomes of decisions regarding the mode of delivery after previous CS [35]. Our results could play a key role in the decision model regarding the mode of delivery at a population level. According to our study, the majority of women initially preferred VD (59.2%). Our results show that most women in this cohort associate VD with the concepts of nature and motherhood (70.3%). A total of 13.6% perceived VD as “better for the body”, “pleasant”, and “less painful”. A total of 8.1% preferred VD due to experiencing a traumatic prior CS. A total of 4.1% of women stated practical reasons, i.e., faster discharge or faster recovery.

The main reasons why women opted for a CS are as follows: traumatic birth experience (27.5%), fear of VD (19.6%), more comfortable (17.6%), fear of complications (9.8%), scar pain (3.9%), shorter interval between pregnancies (3.9%), herniated disc (2%), wish of sterilization during CS (2%), history of myoma enucleations (2%), epilepsy (2%), advanced maternal age (2%), and reason not specified (7.8%).

If we take a closer look, 56.9% of the reasons are related to fear and trauma. Experiencing childbirth can have profound psychological effects, and health professionals should provide dedicated support for women recovering from previous traumatic births.

Factors relating to practicability or convenience (such as comfort, planning for following pregnancies, or concurrent sterilization procedures) account for 23.5% of the reasons for preferring CS.

A total of 9.9% of the reasons for choosing a CS are related to health problems. In this case, it is difficult to say if these are of real concern for the well-being of the patients during and after pregnancy. In obstetrical practice, the emphasis has been on the short-term maternal outcomes of CS [36].

Although maternal age represented a reason for choosing CS, we found no significant difference between the ages of women who preferred VD and those who preferred CS.

A special mention must be attributed to the fear of complications, representing 9.8%. This is the part where the obstetrician or the midwife can play an active, important role. As we can notice from our data regarding the 72 women who decided upon VD after birth planning, twenty underwent CS by necessity and five women delivered per vacuum extraction. Only one of the CSs had the indication of suspicion of uterine rupture, while the majority of the indications were in order of frequency as follows: pathological CTGs, stalled labour, intra-amniotic infection, and unsuccessful labour induction. The five vacuum extractions were indicated by pathological CTGs and only one was complicated with a third-degree tear. For VD, the most frequent complication was the pathological CTG and, in order, green amniotic fluid, nuchal cord, or two or more of these complications.

Open questions remained incomplete for a minority of questionnaires without specification. This could be attributed to social pressure, fear of being judged, shame, or no wish to give a concrete answer.

In total, seventy-eight patients in our study received CS, twenty-eight of them by necessity and fifty through planned CS, seven of which switched from spontaneous birth after the prenatal check-up to planned CS (Table 2).

VD after CS is possible and the main condition for having a safe delivery is to have a well-monitored labour. According to the literature, 24–28% of attempted VDs after CS fail and up to 10% of women scheduled for an elective CS experience spontaneous labour before their planned procedure [35]. In our study, two women who wished for a CS gave birth naturally, without complications. For the first patient, the labour was induced because of stillbirth and the second patient presented to the hospital too late for CS.

We noticed that for many patients who underwent CS by medical necessity, although they tried to deliver vaginally, the indication for the current CS was similar to the indication for the first CS (Table 6). The main indications were failure to progress in labour and/or pathological CTG. Is this a sign that VD for this small group of women is not possible?

Apgar scores at 1 min after birth lower than 8 were registered only in nine cases (three cases of delivery through planned CS; five cases of delivery through CS performed during labour by medical necessity and one vacuum extraction). The lack of a significant difference in Apgar scores at 5 min between VD and CS suggests that the mode of delivery had no impact on the overall well-being of the newborns. We noticed that the maternal and fetal complications registered in our study are similar to the actual literature reports [36,37].

According to the German guidelines, the success rate of vaginal birth after CS is about 75% (60–85%). The same guidelines report a risk of 1% for uterus rupture, 0.1% for hysterectomy, 1.2% for blood transfusion, and 1.2% endometritis during vaginal birth after CS [38].

Reducing the number of CSs would also be achievable if the hospital policy refuses to give the possibility of choice and takes responsibility for the litigation of the doctor or midwife. Constraining women to give birth in an imposed way is an interference with women’s rights and should be up for debate. When women are determined to have CS, they are willing to search for hospitals and doctors who will accommodate their choice, even if it involves additional costs [26].

Women who want to try giving birth vaginally have more positive associations with the process of delivery than women who choose CS. A total of 56.9% of women who preferred CS connected vaginal birth with fear and trauma. According to the literature, mental support during pregnancy plays an important role in improving the mothers’ health, improving levels of attitude, and promoting a positive attitude towards VD [39].

Our study is consistent with the literature, which reports that the majority of women undergo their preferred mode of delivery shortly before their due date [35].

## 5. Conclusions

VD is a real option for patients with a history of CS. The patient must be well informed about the risks and benefits of the medical situation and should be empowered and supported in their chosen mode of delivery, which should be respected.

In our experience, most women arrive for childbirth with a predetermined mode of delivery, and this decision is often made prior to and independently of the medical consultation.

## Figures and Tables

**Table 1:** Preferred mode of delivery before and after the prenatal check-up.

		Preferred Mode of Delivery after Birth Planning		Total
		VD	CS	
Preferred mode of delivery before birth planning	VD	72	2	74
	CS	0	51	51
Total		72	53	125

CS = caesarean section; VD = vaginal delivery.

**Table 2:** Preferred mode of delivery after the prenatal check-up versus the actual mode of delivery.

Preferred Mode of Delivery after Birth Planning	Actual Mode of Delivery				Total
	VD		CS		
	Natural	Vacuum Extraction	Planned CS	CS Performed during Labour by Medical Necessity	
VD	40	5	7	20	72
CS	2	0	43	8	53
Total	42	5	50	28	125

CS = caesarean section; VD = vaginal delivery.

**Table 3:** Indication for current CS.

Indication for Current CS	Planned CS	CS Performed during Labour by Medical Necessity	Total
At the request of the patient with a history of CS	44	10	54
Pathological CTG	0	7	7
Stalled active labour phase	0	5	5
Stalled early labour phase	0	1	1
Macrosomia	2	0	2
Breech presentation	2	0	2
Occiput posterior position	0	1	1
Intra-amniotic infection	0	1	1
Unsuccessful labour induction	0	1	1
Inguinal pain	0	1	1
Suspicion of uterine rupture	0	1	1
History of myoma enucleation	1	0	1
Epilepsy	1	0	1
Total	50	28	78

CS = caesarean section; CTG = cardiotocography.

**Table 4:** Indication for the actual mode of delivery in CS patients who preferred CS after the prenatal check-up.

Indication for the Actual Mode of Delivery in CS Patients Who Preferred CS	Planned CS	CS Performed during Labour by Medical Necessity	Total
At the request of the patient with a history of CS	38	7	45
Breech presentation	2	0	2
Macrosomia	1	0	1
Inguinal pain	0	1	1
History of myoma enucleation	1	0	1
Epilepsy	1	0	1
Total	43	8	51

CS = caesarean section.

**Table 5:** Indication for actual mode of delivery in CS patients who preferred VD after the prenatal check-up.

Indication for Actual Mode of Delivery in CS Patients Who Preferred VD	Planned CS	CS Performed during Labour by Medical Necessity	Total
At the request of the patient with a history of CS	6	3	9
Pathological CTG	0	7	7
Stalled active labour phase	0	5	5
Stalled early labour phase	0	1	1
Occiput posterior position	0	1	1
Intra-amniotic infection	0	1	1
Macrosomia	1	0	1
Unsuccessful labour induction	0	1	1
Suspicion of uterine rupture	0	1	1
Total	7	20	27

CS = caesarean section; CTG = cardiotocography; VD = vaginal delivery.

**Table 6:** Indication for current CS performed due to medical necessity versus indication for prior CS in patients who preferred VD after the prenatal check-up.

		Indication for Current CS Performed due to Medical Necessity				Total
		Pathological CTG	Failure to Progress in Labour	Patient’s Preference	Other	
Indication for Prior CS	Pathological CTG	3	4	0	1	8
	Failure to progress in labour	3	1	0	2	6
	Patient’s preference	0	0	2	0	2
	Other	1	1	1	1	4
Total		7	6	3	4	20

CS = caesarean section; CTG = cardiotocography.

## Data Availability

Further information concerning the present study is available from the corresponding author upon reasonable request.
